# Virtual reality technology enhances the cognitive and social communication of children with autism spectrum disorder

**DOI:** 10.3389/fpubh.2022.1029392

**Published:** 2022-10-06

**Authors:** Junqiang Zhao, Xinxin Zhang, Yi Lu, Xingyang Wu, Fujun Zhou, Shichang Yang, Luping Wang, Xiaoyan Wu, Fangrong Fei

**Affiliations:** ^1^Department of Children Rehabilitation, First Affiliated Hospital of Xinxiang Medical University, Xinxiang, China; ^2^Xinxiang Key Laboratory of Medical Virtual Reality and Augmented Reality, Xinxiang, China; ^3^Xinxiang Intelligent Image Diagnosis Engineering Technology Research Center, Xinxiang, China; ^4^Department of Nursing, Xinxiang Medical University, Xinxiang, China; ^5^Department of Medical Engineering, Xinxiang Medical University, Xinxiang, China; ^6^Department of Psychiatry, The Second Affiliated Hospital of Xinxiang Medical University, Xinxiang, China; ^7^Department of Nursing, Huzhou Maternal and Child Health Care Hospital, Huzhou, China; ^8^Zhejiang Provincial Center for Disease Control and Prevention, Hangzhou, China

**Keywords:** autism spectrum disorder, virtual reality, cognition, social communication, developmental ability, nursing information

## Abstract

**Objective:**

We aimed to explore the impact of using virtual reality technology to intervene in and encourage the developmental behavior areas of cognition, imitation, and social interaction in children with autism spectrum disorder.

**Methods:**

Forty-four children with autism spectrum disorder were divided randomly into an intervention group and a control group, with each group consisting of 22 participants. Incorporating conventional rehabilitation strategies, virtual reality technology was used with the intervention group to conduct rehabilitation training in areas including cognition, imitation, and social interaction. The control group was provided conventional/routine clinical rehabilitation training. The children's cognitive development was evaluated before and 3 months after intervention.

**Results:**

After intervention, the developmental abilities of both groups of children in the areas of cognition, imitation, and social interaction were improved over their abilities measured before the intervention (*P* < 0.05). However, post-intervention score differences between the two groups demonstrated that the intervention group levels were better than the control group levels only in the areas of cognition and social interaction (*P* < 0.05).

**Conclusion:**

Combining virtual reality with conventional rehabilitation training improved the cognitive and social development of children with autism spectrum disorder and supported the goal of improving the rehabilitation effect.

## Introduction

Autism Spectrum Disorders (ASD), also known as autism, is a serious neurodevelopmental disorder that occurs in early childhood and affects five times as many boys as girls. Its clinical manifestations are characterized by unconventional social interaction, narrow interests, and rigid repetition (1). In 2018, 1 in every 59 children aged 4 years in the United States had ASD (2). In China, from 2014 to 2016, the incidence rate of children aged 6 to 12 years living in eight representative cities in China was estimated to be 0.7% (3). A common problem here is factors including the limited number of local and national rehabilitation facilities, a scarcity of practitioners, and different levels of rehabilitation training offered to children with ASD (4). Therapists must spend considerable time and energy creating rehabilitation training scenarios for children with ASD, and clinical treatment has the disadvantages of lengthy rehabilitation or therapy protocols and scenarios that cannot be replicated (5). Therefore, it is necessary to seek additional intervention methods that are more convenient and accessible. Virtual reality (VR) is capable of fusing the actual and virtual worlds, and can replicate various scenarios to produce an immersive experience. A growing number of studies have revealed that VR-based training can improve the outcomes of conventional therapy for patients with ASD (6), and many of these studies have been applied in psychology and development medical therapies (7, 8). VR technology can be utilized to create virtual rehabilitation scenarios and safe, controllable, and repeatable rehabilitation training modes that improve functional development in the social and cognitive abilities of children with ASD. In this study, VR technology was employed in rehabilitation therapies for children with ASD in the areas of cognition, imitation, and social interaction.

## Materials and methods

### Selection of research participants

A total of 47 children diagnosed with ASD in the Pediatric Rehabilitation Department of a Grade A Hospital in Xinxiang from August 2020 to March 2021 were selected as the study's research participants. Patients participating in the intervention were selected according to their admission order. The researchers numbered the patients according to the time of admission. Patients with odd numbers were included in the intervention group, and patients with even numbers were included in the control group. Inclusion criteria included the following:

1. Caregivers with normal cognitive and reading abilities who agreed to participate in the study;
2. Caregivers who were relatives of the children and were aged 20–59 years;
3. Children aged 3–6 years who were stable and conscious patients;
4. Children diagnosed with ASD according to the diagnostic criteria of the Diagnostic and Statistical Manual of Mental Disorders, Fifth Edition (DSM-V).

Exclusion criteria included the following:

1. Children diagnosed with other serious organic diseases;
2. Children and caregiver who were participating in other investigations or interventions;
3. Participants or caregivers who refused to participate or withdrew at any stage of the study;
4. Other serious physical or mental diseases within the family.

### Grouping of research participants

The 47 children who met the inclusion criteria were divided into an intervention group and a control group. In the intervention group, 22 children finished the trial, while 3 dropped out for private reasons; 22 children from the control group finished the research. A total of 44 children with ASD finished the trial.

The intervention group included 19 males and 3 females. Participants were 3–5 years old, with an average age of 3.45 (3.00–4.00) years. Treatment duration was ≥12 months for 4 patients and <12 months for 18 patients. The control group included 16 males and 6 females. The average age of the participants, who ranged in age from 3 to 5 years old, was 3.50 (3.00–4.00). Treatment duration was ≥12 months for 5 patients and <12 months for 17 patients. There were no significant differences in age, gender, or treatment duration between the two groups (all *P* > 0.05). All children and caregivers provided written informed consent. The study was approved by the Ethics Committee of Xinxiang Medical University.

### Methods

#### Intervention team

The intervention team was comprised of five members, one of whom was a pediatric clinician with the title of deputy chief physician. There were two clinical therapists and two nurses, one of whom was a nursing graduate student, who were responsible for data collection and the virtual intervention with the children. Another nurse who had received training from the Heep Hong Association performed the entire evaluation procedure, offered expert analysis of the results, and offered reliable conclusions. The study design for the project involved all team members. Pediatricians were largely in charge of disease diagnosis and providing a rehabilitation treatment plan, and therapists and nurses delivered rehabilitation training and advise in accordance with the pediatricians' recommendations.

#### Intervention methods and content

The control group took part in conventional rehabilitation training, which included the following:

1. Collective class training (40–45 min): The therapist issued oral instructions to the children and waited for the children to respond. If a child responded correctly, reinforcement was given. If the child did not respond appropriately, the therapist paused and then re-issued the instruction, assisted the child, offered a reinforcement, paused again, and then re-issued the instruction once again. If the child did not respond, the therapist assisted the child as necessary.
2. Sensory integration training (40–45 min): This training utilized cylinders, a wooden balance table, push balls (to be used on the ground), horn balls (tactile exercise equipment), and other touchdown training tools. The children used the equipment to use gross motor skills for balance and defense training in which different parts of the body had to operate harmoniously and effectively. Classes were held each day, Monday through Friday, and two types of tools were used in each class. The instructor made adjustments according to each child's progress and current developmental level, which was assessed every week.
3. Fine motor training (40–45 min): This training included manual tasks based on the child's specific ASD diagnosis and abilities. Each week the children's training included picking up items, panel matching, and similar tasks. These manual tasks aided the developed the children's sensory organs, including their eyes and ears, helping to develop the functionality of their brains and body parts and the coordination of their physical and cognitive abilities.

In the intervention group, VR technology was added to the rehabilitation training offered to the control group. In the virtual scene, children were instructed to hold a cursor on a target or to answer questions according to the instructions given by virtual characters across different scenes to proceed to the next step. During the intervention, several children refused to wear head mounted VR displays. Considering the characteristics of patients with ASD, “desensitization therapy” was adopted 1 week before the intervention for children who resisted the rehabilitation training. Play time was increased as needed for each child until the child was able to accept and participate in the intervention program. In the intervention phase, each child received intervention training three times a week. Each training session totaled 15 min (3 instances of 5 min of training, with rest periods in between). The intervention period was a total of 12 weeks.

**Figure 1:**
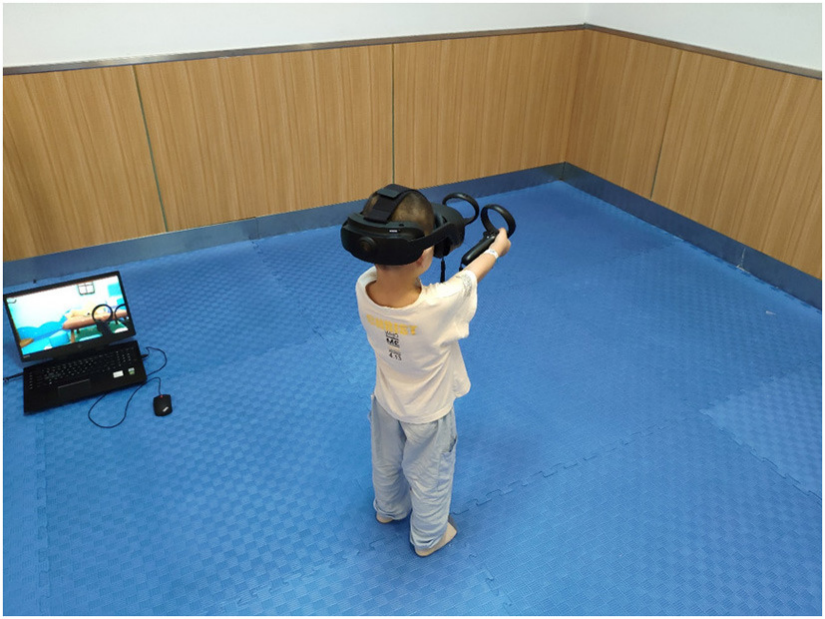
Children in the intervention group were trained with virtual reality technology.

Researchers, clinicians, and therapists designed the VR training based on an accepted rehabilitation intervention method for the ASD population: applied behavior analysis (9). For example, if a child could not complete object identification after the virtual teacher had given instructions, the target object would flash to replace the teacher's assistance in real-world training. The virtual scene was designed considering the clinical characteristics of children with ASD, in that the advantages of picture communication, visual stimulation, and cartoon animation were combined. Unity3D was used to develop the VR training. The game was divided into six training scenes focused on the cognitive, social, imitation, gross motor, emotional expression, and language understanding of children with ASD, as shown in Table 1. Figure 1 shows a child participating in the VR training.

**Table 1:** Six virtual reality intervention training scenarios.

**Scene**	**Image from the scene**	**Tasks**	**Focus area**
Scene 1: Looking for things	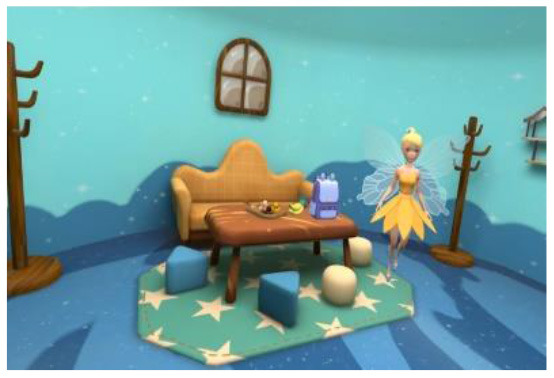	Look for bags, maps, bananas, etc.	Cognitive training
Scene 2: The garden	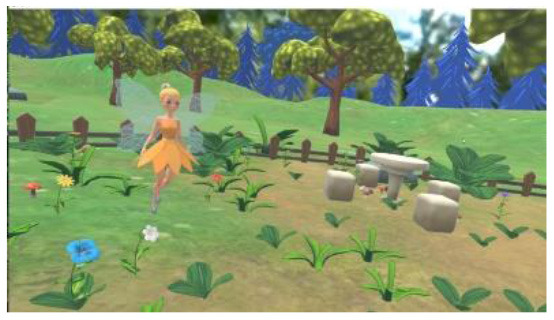	Find flowers of the correct color and place them in the correct basket	Cognitive training
Scene 3: Forest animals	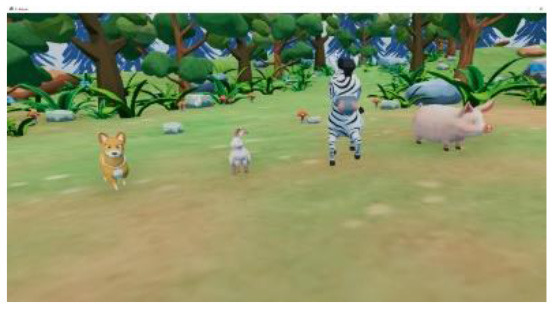	Look for small animals and get to know the height	Emotional expression
Scene 4: Crossing the river	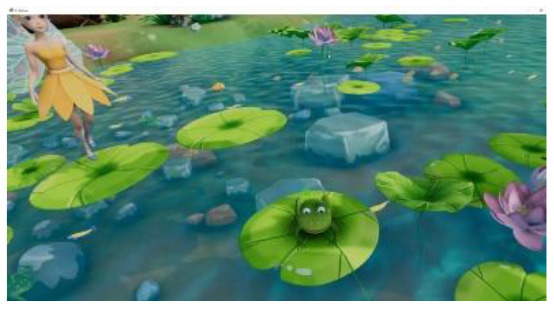	Say hello to small animals and communicate verbally	Social practice; Gross motor training
Scene 5: Get to know your vegetables	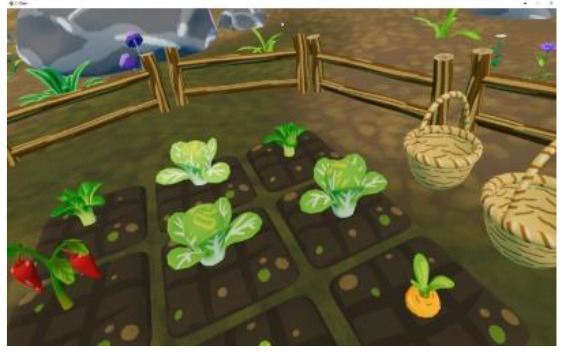	Classify vegetables and imitate the actions of virtual characters	Imitation training
Scene 6: Selling vegetables	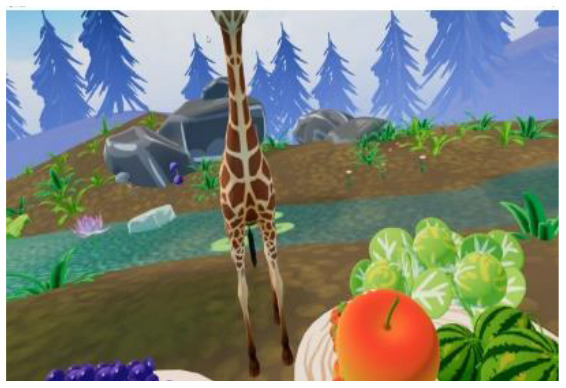	Identify vegetables and consolidate learning	Language understanding

#### Evaluation methods

The Psychoeducational Profile, Third Edition (PEP-3) was used to evaluate the rehabilitation impact on children with ASD before and after intervention. The Chinese version of the PEP-3 assessment is divided into three parts: developmental and behavioral side tests, child caregiver reports, and composite scores. The developmental and behavioral side tests include 34 assessments of cognitive behaviors, 25 assessments of verbal expression, 19 assessments of verbal comprehension, 20 assessments of fine motor skills, 15 assessments of gross motor skills, 10 motor imitation tests, 11 emotional expression tests, 12 social interaction tests, 15 non-verbal behavior tests, and 11 verbal ability tests. The child caregiver report includes 10 queries about problem behaviors, 13 queries about self-care behaviors, and 15 queries about adaptive behaviors. This study used six development areas in the development and behavioral subtest: cognitive, language comparison, cross motor, mimicry, social interaction, and emotional expression.

The PEP-3 can be used to evaluate the strength and development/adaptation of children with ASD. Individual scores for each development and behavioral side test are expressed as 0 (fail), 1 (intermediate response), or 2 (pass) points. The corresponding age and percentage progression of children's development parts can be found through the original score. A percentage progression >89 indicates appropriate degree of development/adaptation; 75–89 indicates a slight degree of development/adaptation; 25–74 indicates moderate degree of development adaptation; and <25 indicates a severe degree of development/adaptation. The Cronbach'α coefficient of the scale is 0.84.

All participants were evaluated before intervention and 3 months after intervention. After obtaining the consent of guardians, nurse evaluators assessed the children and completed the evaluation questionnaire, which was collected immediately. A total of 47 questionnaires were issued; 44 questionnaires were recovered effectively, with a questionnaire recovery rate of 94%.

#### Statistical analysis

SPSS 25.0 software was used for statistical analysis of the data. If the data followed normal distribution, a two-sample/independent *t*-test would be used for inter-group comparison. A paired *t*-test was used to compare results before and after intervention. Non-normal distribution data were tested by a non-parametric rank-sum test, with a test level α = 0.05. For the data followed normal distribution, we display mean ± SD, for the data not followed normal distribution, we display quartile.

## Results

### Comparison of scores across various abilities before intervention

Before the intervention, there was no statistical difference between the cognitive development scores of the two groups (*P* > 0.05), as shown in Table 2.

**Table 2:** Score comparison of the two groups before intervention.

**Group/Statistics**	**Cognitive**	**Language comprehension**	**Gross motor**	**Mimicry**	**Social interaction**	**Emotional expression**
Intervention Group (*n* = 22) score	30.50(21.75~36.00)	19.50(13.50~23.00)	27.00(24.75~28.00)	12.64 ± 2.72	11.18 ± 2.40	9.95 ± 3.96
Control group (*n* = 22) score	20.50(13.00~35.00)	13.00(9.75~21.25)	27.00(24.75~29.00)	11.23 ± 3.68	10.36 ± 2.87	7.95 ± 2.95
t/Z	−1.657	−1.624	−0.202	1.446	1.025	1.900
*p*	0.098	0.104	0.840	0.156	0.311	0.064

### Comparison of scores across various abilities after intervention

In both groups, the post-intervention developmental ability scores in the areas of cognition, imitation, and social interaction were significantly better than the pre-intervention scores (*P* < 0.05; see Table 3). This result indicates that the intervention program was effective in improving the ability of children with ASD.

**Table 3:** Score comparison of the two groups before and after intervention.

	**Intervention group (*****n*** = **22)**				**Control group (*****n*** = **22)**			
**Domain**	**Before intervention**	**After intervention**	**t/Z**	**p**	**Before intervention**	**After intervention**	**t/Z**	***p***
Cognitive	29.86 ± 9.47	33.59 ± 9.38	−12.309	0.000	20.50(13.00~35.00)	21.50(13.00~35.25)	−2.456	0.014
Language	18.41 ± 6.29	20.86 ± 6.40	−8.860	0.000	13.00(9.75~21.25)	16.00(11.00~23.25)	−4.131	0.000
Gross motor	25.00(23.00~27.00)	26.50(24.00~29.00)	−4.104	0.000	25.00(24.00~27.00)	26.50(25.00~28.00)	−4.011	0.000
Mimicry	12.64 ± 2.72	14.45 ± 2.65	−10.727	0.000	11.23 ± 3.68	12.73 ± 3.67	−5.936	0.000
Social interaction	11.18 ± 2.40	13.59 ± 2.15	−14.189	0.000	10.36 ± 2.87	11.73 ± 2.75	−12.990	0.000
Emotional expression	9.95 ± 3.96	10.95 ± 3.75	−5.066	0.000	7.95 ± 2.95	8.50 ± 2.84	−5.020	0.002

### Comparison of score difference between two groups after intervention

After the intervention, the cognitive ability and social communication ability of the children in the intervention group were significantly higher than in the control group (*P* < 0.05; see Table 4). These results indicate that VR-assisted intervention technology can promote the development of these abilities in children with ASD.

**Table 4:** Score comparison of the two groups before and after intervention.

**Group/Statistics**	**Cognitive**	**Language comprehension**	**Gross motor**	**Mimicry**	**Social interaction**	**Emotional expression**
Intervention group (*n* = 22) scores	3.50(3.00^~^5.00)	2.00(1.75^~^3.25)	1.50(1.00^~^2.00)	2.00(2.00^~^3.00)	2.00(1.00^~^2.00)	1.00(0.00^~^2.00)
control group (*n* = 22) scores	0.00(0.00^~^1.00)	2.00(1.00^~^3.00)	1.00(1.00^~^2.00)	1.00(1.00^~^2.00)	1.00(1.00^~^2.00)	1.00(0.00^~^1.00)
t/Z	−5.173	−0.678	−0.484	−4.233	−1.617	−1.642
*p*	0.000	0.498	0.629	0.000	0.106	0.101

## Discussion

### Rehabilitation therapy based on VR technology is beneficial to the cognitive developmental ability of children with ASD

The results of this study demonstrated that there was no significant differences in the developmental ability scores of the children in the control and intervention groups before the intervention across gender, age, treatment time, and cognition variables (*P* > 0.05). After 3 months of rehabilitation, the intervention group's developmental ability scores for cognition, imitation, and social interactions were significantly higher than before the intervention (*P* < 0.05). The scores of the children in the intervention group were also significantly better than the scores of the children in the control group for cognition and social interaction abilities (*P* < 0.05). The study results suggest that both conventional clinical rehabilitation training and conventional rehabilitation training with VR training can affect the developmental ability of children with ASD (10–12). These results confirm results from previous studies (13).

Patients with ASD often have anxiety and fear about unfamiliar environments (14–17). Frequent staffing changes occur within rehabilitation teams, and children with ASD often do not cooperate, which can lead to potential delays in the rehabilitation process and increasing difficulty for medical personnel (18–20). A VR environment has the advantages of safety, controllability, and repeated operation. Its immersive picture makes children with ASD receptive to visual stimulation, which is conducive to cognitive learning (21–25). Moreover, social stories are presented in the form of virtual animation, and it tends to be simple to interest and immerse children in animation and to maintain their attention for a long time (26, 27). With these advantages, the use of virtual training can help further improve children's cognitive ability.

### Rehabilitation therapy based on VR technology is beneficial to the development of social communication ability for children with ASD

One core issue in ASD is that social communication is very difficult (28–30). The results of this study show that the rehabilitation effect in terms of social interaction improved for the children in the intervention group. Some nonverbal communication skills, such as greeting, were included in the study's VR training. Additionally, in the virtual scenes, the tasks presented to the children had them face and learn about emotional reactions in social interaction. Under the supervision of nurses, social skills learned by children in the training environment (including facial expression recognition, happy tone of voice, and body language based on the actions of virtual characters) can be generalized into daily life gradually. This integration mobilizes overall mental state, which can increase the enthusiasm of children in rehabilitation training (31). Adopting multi-level and multi-channel VR social training can further improve children's social ability.

### Limitations

#### Small sample size

One limitation of this study was its small sample size. The sample size should be increased in future studies to track and record the developmental ability of children with ASD to further confirm the applicability and value of this study.

#### Curative effects

For abilities other than cognitive development and social communication, although VR technology can produce dynamic interaction, skill levels and the development obtained in specific training contexts must be further tested and trained in actual social situations (32–34). The timeframe of this study was short, with only 3 months of intervention. Moreover, whether the training described in this study can build on rehabilitation effects in other fields is a research question deserving further discussion and investigation.

#### Training guidance

In the virtual environment, it is difficult for children to control their behavior and explain the reasoning or purpose behind their choice of behavior. In this study, the nurses could not enter the virtual environment to help the children understand the situation and guide them to make appropriate responses (35). This restriction may also limit children's use of VR technology for rehabilitation training of related skills. If the nurse and the child could appear together in the scene and perform task decomposition through voice instructions, the rehabilitation effect may be better. However, current technology levels make it difficult to solve this problem.

#### Patient compliance

Children with ASD typically demonstrate social withdrawal, anxiety, and fear in unfamiliar environments. Training with VR requires long periods of concentration, which is a challenge for these, and many, children. In this study, it was feasible for the nurses to use items of interest to the children (i.e., “reinforcement”) to increase the children's compliance during the training. The question of how to optimize the volume, pictures, scene-switching mode, and more within VR to produce more positive effects for the children must be considered.

## Conclusion

Many studies have found that VR training helps children with ASD and patients with adjacent neurological diseases to build various skills, such as language function, attention, and executive function (36, 37). Our study found that rehabilitation training based on VR technology can effectively promote the cognitive and social communication abilities of children with ASD. This study's results offer a new and meaningful rehabilitation method for the ongoing clinical rehabilitation of children with ASD. However, no significant differences were seen between the children in the control and intervention groups for areas of development other than cognition and social communication.

## Data availability statement

The raw data supporting the conclusions of this article will be made available by the authors, without undue reservation.

## Ethics statement

The studies involving human participants were reviewed and approved by Ethics Committee of Xinxiang Medical University. Written informed consent to participate in this study was provided by the participants' legal guardian/next of kin.

## Author contributions

JZ, XZ, and YL analyzed the data and drafted the manuscript. XinW, FZ, SY, and LW analyzed the data and designed the VR environment. XiaW and FF designed the research and revised the manuscript. All authors agreed to be accountable for the content of the work.

## Funding

The work was financially supported the Key R&D and Promotion Projects in Henan Province (222102310615), Henan Province Medical Science and Technology Key Project Jointly Constructed by Province and Ministry (SBGJ202102189), and Research on Visualization Construction of TAVR Therapy Based on Virtual Reality Technology (XZZX2022011).

## Conflict of interest

The authors declare that the research was conducted in the absence of any commercial or financial relationships that could be construed as a potential conflict of interest.

## Publisher's note

All claims expressed in this article are solely those of the authors and do not necessarily represent those of their affiliated organizations, or those of the publisher, the editors and the reviewers. Any product that may be evaluated in this article, or claim that may be made by its manufacturer, is not guaranteed or endorsed by the publisher.
